# Can a hybrid chemical-ferromagnetic model of the avian compass explain its outstanding sensitivity to magnetic noise?

**DOI:** 10.1371/journal.pone.0173887

**Published:** 2017-03-15

**Authors:** Kirill Kavokin

**Affiliations:** 1 Spin Optics Laboratory, St. Petersburg State University, St. Petersburg, Russia; 2 I.M.Sechenov Institute of Evolutionary Physiology and Biochemistry RAS, St. Petersburg, Russia; Syddansk Universitet, DENMARK

## Abstract

While many properties of the magnetic compass of migratory birds are satisfactory explained within the chemical model of magnetoreception, its extreme sensitivity to radio-frequency magnetic fields remains a mystery. Apparently, this difficulty could be overcome if the magnetoreceptor model were augmented with a magnetite nanoparticle, which would amplify the magnetic field at the position of the magneto-sensitive cryptochrome molecule. However, comparison of the radio-frequency power used in the experiment with intrinsic magnetization noise of such a particle, estimated from the theory of fluctuations, shows that the required sensitivity cannot be reached with realistic parameters of iron-oxide nanocrystals.

## 1. Introduction

The magnetic sense (magnetoreception) and its use by various animals for orientation and navigation is one of the hot topics of research in sensory physiology and behavioral zoology [1]. In recent years, it was realized that magnetoreception also presents interesting and not easy to solve problems for physicists and chemists. Though the existence of the magnetic sense in many animals was proved by numerous experiments, the receptor (or receptors, because supposedly there exist at least two different kinds of such) was not reliably demonstrated for any species of higher animals.

Nevertheless, in one case, namely the compass receptor of migratory birds, non-trivial quantitative information was obtained in a series of intricate behavioural tests. This experimental approach, designed and realized in the group of R. and W. Wiltschko in Frankfurt [2–5], was at some stage called “behavioural spectroscopy”. By applying to a bird in the test cage a weak magnetic field oscillating at the frequency close to electron spin resonance in the static ambient field, the experimentalists were able to disrupt the bird’s ability to orient. The disruptive effect of the oscillating field was found to be sensitive to its frequency; hence this method can indeed be called spectroscopy.

Initially, these experiments were planned to test one of the most popular theories of magnetoreception, the radical pair model (RPM) [6–8]. RPM relies on magnetic sensitivity of photochemical reactions in a protein called cryptochrome, accompanied by formation of a pair of radicals carrying unpaired electron spins. The outcome of such a reaction depends on preservation of the initial correlation between electron spins in the radicals: while remaining in the singlet state, the radicals have a large probability to recombine, but upon conversion into the triplet recombination is blocked, and each of the radicals obtains a chance to enter a chain of chemical transformations that may start transduction of a neural signal. With spin relaxation times of electrons in the radical pair of the order of few microseconds, interconversion between singlet and triplet spin states can be affected by static magnetic fields comparable in strength to the geomagnetic field [9,10]. One can suggest that the reaction outcome can change also under influence of weak radio-frequency fields when the frequency of the perturbation is close to the electron spin resonance (ESR) of one of the two spins. The other mainstream theory of magnetoreception, involving ferromagnetic or ferrimagnetic nanocrystals as sensitive elements [11], is presumed unable to explain this effect, because fast spin-lattice relaxation in relevant iron-oxide magnetic nanoparticles does not allow the weak RF field to deflect the particle magnetic moment from its equilibrium direction. A possibility of heating the nanocrystals by the RF field was theoretically discussed [12]; however, it requires long heat accumulation times, of the order of hours, and very good thermal isolation of the magnetoreceptor, which does not look realistic. Therefore, the discovery of the RF field effect on the birds’ compass was interpreted as a strong argument in favour of the radical pair mechanism as a physical basis for orientational magnetoreception.

A quantitative analysis [13] showed, however, that this optimistic conclusion was premature: in fact, the RF field amplitudes reported to disrupt orientation of caged European robins (*Erithacus rubecula*) were too small to cause a considerable change of the yield of RP reactions. The overall reason is that the rate of spin transitions caused by so weak a field (15 nT monochromatic or 85 nT broadband fields) is much slower than spin relaxation in organic radicals. For example, the transition rate of an electron spin under the broadband “noise” field in the frequency range from 0.1 to 10 MHz with the integral rms amplitude of 85 nT [2] was estimated as 1 per second [13]. With spin lifetimes in organic radicals typically not exceeding few microseconds, so weak a perturbation is not expected to induce any considerable change to population of spin levels as well as to spin-spin correlation. The outstanding sensitivity of the birds’ compass attracted attention of many theoreticians (see, for example, [14–16]) who tried to explain it within RPM, invoking various quantum and statistical effects—without a decisive success. A careful discussion of this problem in [17] ended up with the conclusion that RPM might need modifications, under the condition that unusual experimental results of the Wiltschkos’ group be independently confirmed.

In recent years, two publications appeared, coming from different laboratories, which reproduced certain results of the Wiltschkos’ group [18,19]. Besides that, new important quantitative results were reported on the effect of broadband RF fields [18]. These findings strongly suggest that the RF field effect is a reality, and the theory of orientational magnetoreception must be able to explain it.

It should be mentioned that there is growing evidence that, in spite of difficulties of RPM with explaining RF field effects, the compass magnetoreceptor might be based on photochemical reactions in cryptochrome [20]. However, the RPM should be further developed to address unresolved or poorly resolved problems. One possible way to do this is to suppose that the magnetic sensor has a hybrid structure, comprising both the cryptochrome molecule and a magnetic particle which would serve as an antenna for amplifying external magnetic fields. Binhi [21] and later Cai [22] suggested that it might enhance the efficiency of magnetoreception; in particular, magnetic field gradients near the magnetic nanoparticle might accelerate singlet-triplet interconversion in a radical pair. Effects of magnetic nanoparticles on radical pair reactions, outside the magnetoreception context, were theoretically considered in [23], and experimentally observed in [24].

The aim of this study is to understand whether such or similar hybrid structure can demonstrate the outstanding sensitivity to radiofrequency magnetic field observed in experiments with migratory birds.

Out of the whole body of experimental data on the RF field effect, reported so far, we select for the quantitative analysis the effect of broadband RF fields [2,18]. The reasons for this are the following:

1. this effect has been independently reported by two research groups;
2. it gives a possibility to estimate transition and/or decoherence rates without a detailed knowledge of the spectrum of electron spin states involved.

## 2. Methods

In this paper we apply the fluctuation-dissipation theorem and the theory of spin relaxation to analyse sensitivity and intrinsic noise of the hypothetical hybrid magnetoreceptor.

The paper is organized as follows: in Section 3.1, latest experimental results on the effect of RF magnetic noise on the birds’ compass [18] are compared with theoretical calculations of spin relaxation times in cryptochrome [25]. In Section 3.2, a hybrid magnetoreceptor model is introduced involving a superparamagnetic nanoparticle, its responses to static and oscillating fields are calculated and compared with the experimental results; in Section 3.3, the intrinsic noise of the relevant magnetic particle is estimated using the fluctuation-dissipation theorem and compared to artificial noise fields applied in experiments; in Section 6, the implications of the results of sensitivity and noise analysis are discussed, as well as possible directions of future experiments.

## 3. Results

### 3.1. Estimation of the effect of noise magnetic fields within the radical pair model

The radical pair in the transient magneto-sensitive state comprises two non-interacting electrons, each coupled to its own environment of nuclear spins [26]. Initially they are in the singlet state. The processes of coherent interconversion and spin relaxation eventually bring the electrons into fully thermalized state where probabilities of triplet and singlet configuration relate as 3:1. External fields, being uniform across the radical pair, interact with the total magnetic moment of the two electrons. If magnetic moments of the two electrons are equal to each other (which is to a good approximation true for organic radicals) external magnetic fields do not cause transitions between singlet and triplet states, which are eigenstates of the total spin operator of the electron pair [27]. The singlet-triplet interconversion can be caused either by a small difference of electron g-factors in the radicals, or, more commonly, by hyperfine interactions, which are generally unequal for the two electrons. The rate of this hyperfine-induced interconversion is affected by uniform external magnetic fields. In some special cases, the effect of external field may strongly depend on its orientation with respect to certain directions within the molecule. For instance, application of an Earth-strength magnetic field perpendicularly to the main axis of hyperfine coupling with nitrogen nuclei in the Flavin radical was shown to considerably decrease the singlet yield in cryptochrome [10].

Spin relaxation processes within the radical pair, induced, for instance, by modulation of hyperfine coupling by thermal motion of radicals [25], usually suppress the sensitivity of the radical pair reaction to magnetic fields (in some special cases a reverse effect is possible [28]). The spin relaxation is a consequence of decoherence and quantum transitions caused by random intra-molecular magnetic fields with finite spectral densities at resonance frequencies of the spin system of the radical pair. One may expect the external magnetic noise to produce an additional effect on the RP reaction yield if its spectral power density $b_{ext}^{2}$ is comparable or larger than that of internal random fields. Calculations, performed in [25] for the plant cryptocrome AtCry, give zero-frequency inverse spectral densities of random fields induced by different nuclei, expressed in time units:

$$J{\left(0\right)}^{-1}={\left[\gamma_{e}^{2}b_{\text{int}}^{2}\left(0\right)\right]}^{-1}$$ (1)

where $b_{\text{int}}^{2}$ is the spectral power density of an internal hyperfine field. The obtained values of *J*(0)^−1^ are of the order of 100ns. Assuming that spectral densities of the hyperfine fields at MHz frequencies can be lower than those at zero frequency, and bird cryptocromes might differ in this aspect from AtCry [25], one might accept the value of 1 microsecond as a reasonable estimate. With the electron gyromagnetic ratio *γ*_*e*_ ≈ 176*rad* / *s*·*nT*, this corresponds to $b_{\text{int}}^{2}\approx 200nT^{2}/Hz$. Experimental data of Ref [18] suggest that the sensitivity threshold of the magnetic compass of European robins to magnetic noise corresponds to the field spectral amplitude of about 1 nT measured within a functional window of 10kHz. This makes $b_{ext}^{2}\approx 10^{-4}nT^{2}/Hz$, that is six orders of magnitude weaker than the spectral power density of internal random fields. With this huge difference, no effect of the external magnetic noise on the yield of the radical pair reaction can be expected.

The question remains open whether the electric component of the broadband field, also present in the experiments of Ref. [18], could have any effect on the radical pair reaction.

### 3.2. Hybrid model: Magnetic permeability issue

**Fig 1:**
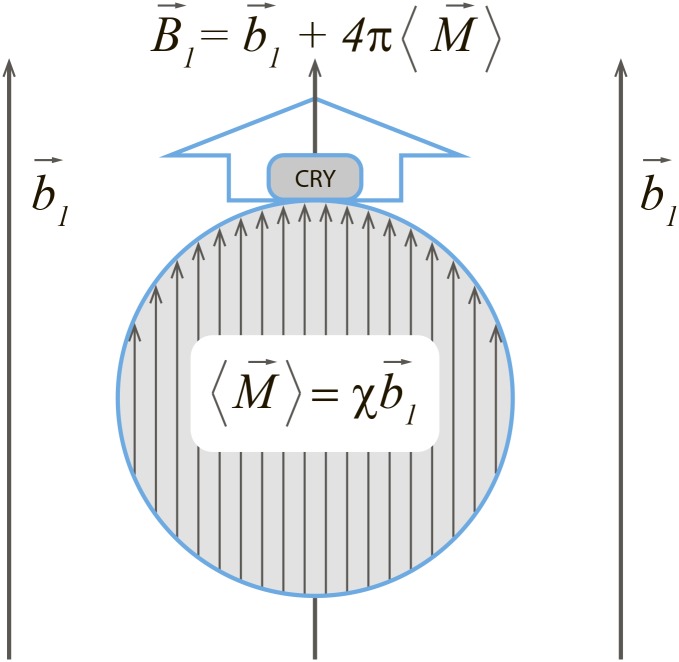
Sketch of a hybrid magnetoreceptor. The magnetic field at the cryptochrome position, *B*_1_ can be strongly amplified if the external field ${\vec{b}}_{1}$ is applied along the line connecting the cryptochrome position and the centre of the particle.

A sketch of the hybrid magnetoreceptor is shown in Fig 1. It consists of a magnetic particle (presumably, an iron oxide nanocrystal) and a closely spaced cryptochrome molecule. The simplest imaginable mode of its operation is that of an iron-core electromagnet: the maximum enhanced field *B*_1_ near the magnetic particle is related to the driving external field *b*_1_, applied as shown in Fig 1, through the relative permeability *k* of the particle:

$$B_{1}\approx kb_{1}$$ (2)

To raise spectral power densities of fields applied to the cryptochrome to the level of 10% of calculated spectral densities of internal random fields [25], the external noise field must be amplified by approximately 300 times, requiring *k* ≈ 300. To compare, relative permeability of magnetically soft steels is measured in hundreds, while *k* of a few thousand is more typical for permalloy. These artificial high-permeability materials are very unlikely to be found in biological systems. Here our choice is restricted to ferrimagnetic iron compounds like magnetite, maghemite or greigite, which in bulk multi-domain form have much lower permeabilities, normally less than 10 [29,30]. But the figures for small superparamagnetic particles can be more favourable. Such a particle [31], comprising just one magnetic domain, would demonstrate the paramagnetic susceptibility, inversely proportional to the absolute temperature *T*, above the blocking temperature *T*_*B*_ (below T_B_ its magnetic moment is frozen parallel to the principal anisotropy axis of the particle):

$$\chi =\frac{d\langle M\rangle }{dB}=V^{-1}\frac{\mu^{2}}{3k_{B}T}=V\frac{M_{S}^{2}}{3k_{B}T}$$ (3)

where V is the particle volume, *μ* = *VM*_*S*_ is its magnetic moment, *M*_*S*_ is the saturation magnetization of the material, 〈*M*〉 is the mean (time-averaged) magnetization in the magnetic field *B*. Here and in the rest of the paper we use the Gaussian (CGS) system of units. Converting *χ* into the relative permeability with the relation

k=1+4πχ (4)

and taking 500G as an upper estimate for saturation magnetization (for instance, it is 470-480G for magnetite and about 240G for greigite), one easily finds out that to provide the needed enhancement of the external RF field the magnetic particle should have the volume not less than approximately1.3·10^−17^ *cm*^3^ (equivalent cube size 23nm). This is reasonably below the boundary between superparamagnetic and single-domain magnetite crystals with small aspect ratio, about 10^−16^ cm^3^ [11, 32] (equivalent cube size 46nm). Therefore, at least in principle, superparamagnetic nanocrystals can provide field enhancement sufficient to explain the effect of RF magnetic field on the avian compass.

### 3.3. Hybrid model: Intrinsic noise issue

Another limiting factor for the hybrid magnetoreceptor is the intrinsic magnetic noise produced by the superparamagnetic particle. Indeed, while the magnitude of the particle’s magnetic moment is maintained constant by the isotropic exchange interaction and is equal to the saturation magnetization of the material times the particle volume, its direction is random and changes all the time because of thermal agitation. Considering the magnetic nanocrystal as a magnetic antenna, one can characterize it, as any device transmitting and/or amplifying analog signals, by its noise equivalent power (NEP). NEP is such power of input signal, which produces, within the 1 Hertz bandwidth, the same output power as the intrinsic noise of the device. NEP of the magnetic particle considered as a magnetic antenna can be directly compared with the threshold sensitivity of the bird compass to broadband RF magnetic fields, which can be estimated from the data of Ref [18]. Indeed, the sensitivity of the hybrid receptor to broadband signals cannot be higher than NEP of its magnetic antenna, because in that case the power of amplified signal acting upon the cryptochrome would be smaller than the noise power created by the antenna.

A superparamagnetic particle has zero average magnetic moment in the absence of external magnetic field. Its magnetic-moment fluctuations are characterized by a correlation function, which, since the magnetic moment is a vector, has the form of a second-rank tensor [33]. Each of the 3 principal components of this tensor decays with certain relaxation time. For instance, the magnetic-moment fluctuations along the main anisotropy axis Z have the following auto-correlation function [33]:

$$\langle \mu_{z}\left(0\right)\mu_{z}\left(t\right)\rangle =\langle \mu_{z}^{2}\rangle \text{exp}\left(-\left|t\right|/\tau \right),$$ (5)

where *τ* is the corresponding relaxation time. This expression is applicable also to the case of isotropic or cubic particle where $\vec{\mu }$ can be directed along all the three Cartesian axes with equal probabilities. The mean squared fluctuation $\langle \mu_{z}^{2}\rangle$ is then equal to one third of the squared magnetic moment of the particle:

$$\langle \mu_{z}^{2}\rangle =\frac{1}{3}\mu^{2}=\frac{1}{3}{\left(M_{S}V\right)}^{2}.$$ (6)

In the opposite extreme case of strong uniaxial anisotropy, $\vec{\mu }$ can be directed either along the anisotropy axis Z or opposite to it, and

$$\langle \mu_{z}^{2}\rangle =\mu^{2}={\left(M_{S}V\right)}^{2}$$ (7)

The spectral power density of *μ*_*z*_ is then obtained as the Fourier transform of Eq (5):

$${\langle \mu_{z}^{2}\rangle }_{\omega }=\int_{-\infty }^{+\infty }\langle \mu_{z}\left(0\right)\mu_{z}\left(t\right)\rangle \text{exp}(i\omega t)dt=\langle \mu_{z}^{2}\rangle \frac{2\tau }{1+\omega^{2}\tau^{2}}$$ (8)

According to the fluctuation-dissipation theorem [33], the imaginary part of the generalized susceptibility *α*_*z*_(*ω*) (a complex-number coefficient through which the induced magnetic moment is related with the applied RF magnetic field; absorbed RF power is proportional to its imaginary part ${\alpha^{\prime \prime }}_{z}\left(\omega \right)$) is connected to the spectral power density of the magnetic moment as

$${\alpha^{\prime \prime }}_{z}\left(\omega \right)=\frac{\omega }{2k_{B}T}{\langle \mu_{z}^{2}\rangle }_{\omega }=\frac{\langle \mu_{z}^{2}\rangle }{k_{B}T}\frac{\omega \tau }{1+\omega^{2}\tau^{2}}$$ (9)

Since real and imaginary parts of *α*_*z*_(*ω*) are connected by the Kramers-Kronig relations [33], the expression for the real part of susceptibility, ${\alpha^{\prime }}_{z}\left(\omega \right)$, reads:

$${\alpha^{\prime }}_{z}\left(\omega \right)=\frac{\langle \mu_{z}^{2}\rangle }{k_{B}T}\frac{1}{1+\omega^{2}\tau^{2}}$$ (10)

In order to calculate NEP of the ferromagnetic particle, one should divide spectral power density of *μ*_*z*_ (Eq (8)) by the squared modulus of generalized susceptibility,

$${\left|\alpha \left(\omega \right)\right|}^{2}={\left[{\alpha^{\prime }}_{z}\left(\omega \right)\right]}^{2}+{\left[{\alpha^{\prime \prime }}_{z}\left(\omega \right)\right]}^{2}={\left(\frac{\langle \mu_{z}^{2}\rangle }{k_{B}T}\right)}^{2}\frac{1}{1+\omega^{2}\tau^{2}}$$ (11)

This way, we obtain a general expression for the equivalent power of magnetic noise $b_{NEP}^{2}$ of an isotropic or weakly anisotropic superparamagnetic particle:

$$b_{NEP}^{2}=\frac{{\langle \mu_{z}^{2}\rangle }_{\omega }}{{\left|\alpha \left(\omega \right)\right|}^{2}}=\frac{2{\left(k_{B}T\right)}^{2}\tau }{\langle \mu_{z}^{2}\rangle }=\frac{2\eta {\left(k_{B}T\right)}^{2}\tau }{M_{s}^{2}V^{2}}$$ (12)

where *η* = 3 for an isotropic particle and *η* = 1 for the case of strong uniaxial anisotropy.

One can see that $b_{NEP}^{2}$ of such a particle does not depend on frequency.

## 4. Discussion

**Fig 2:**
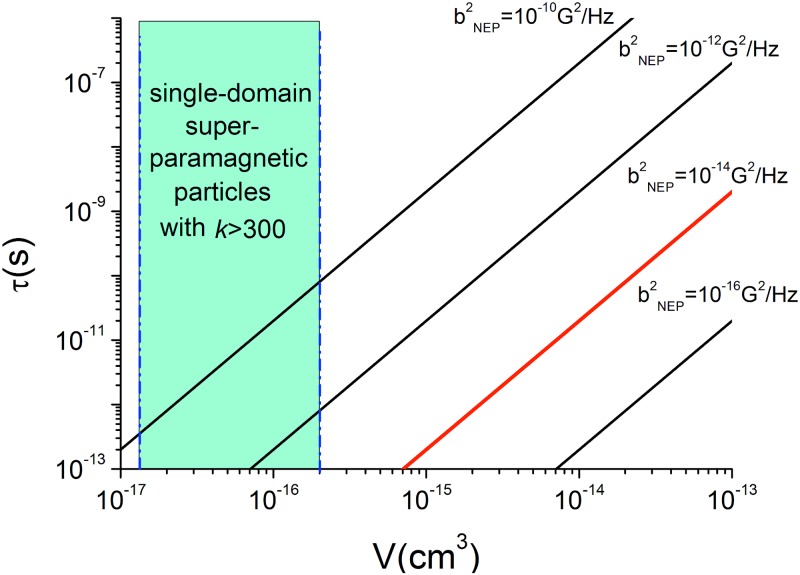
The noise equivalent power $b_{NEP}^{2}$ of magnetic fluctuations as a function of volume and magnetization relaxation time of an isotropic magnetite particle. The blue area shows the region where the particle can remain superparamagnetic at biological temperatures and provide the relative permeability *k*>300. The area to the right of the red line corresponds to the magnetic noise powers at which European robins were disoriented, according to Ref. [18].

Fig 2 shows, on the *V*–*τ* plane (where *V* is the volume of the magnetic particle, and *τ* is the relaxation time of its magnetic moment), the restrictions imposed on the particle in question by the observed sensitivity of the bird compass to broadband magnetic noise.

One can see that the required amplification factor 300 expressed via the relative permeability *k* can be easily realized with a superparamagnetic magnetite particle.

If we follow the reasoning of Section 3.3, and postulate that the noise equivalent power of the antenna of the hybrid receptor should not be higher than the threshold power density of the broadband magnetic field that caused disorientation of European robins in the experiments of Ref. [18], we shall face much more severe restrictions on the parameters of the magnetic particle serving as the antenna. The data of Ref. [18] suggest the threshold power density of about 10^−14^ *G*^2^ / *Hz*; the region on the *V*–*τ* plane, corresponding to that low $b_{NEP}^{2}$, does not overlap with the region where superparamagnetism theoretically can exist in perfectly isotropic magnetite particles [11, 32], even if the particle has an extremely short relaxation time (to the best of my knowledge, the shortest time *τ* reported for magnetite nanoparticles was 1.3·10^−12^ *s* [34]; more realistic values of relaxation times are of the order of 1ns [35, 36]). These considerations suggest that superparamagnetic iron-oxide particles are rather not suitable as magnetic antennas for the hybrid magnetoreceptor; at least, such a receptor cannot demonstrate the observed sensitivity to magnetic noise. It is not clear whether more complex, multi-domain or multi-grain structures can be designed in such a way as to overcome these restrictions.

It should be noted, however, that interpretation of the data of Refs. [18, 37] in terms of spectral power density of magnetic noise is not straightforward because of the measurement method used (in some experiments, maximum field amplitude during certain measurement time was recorded rather than the power spectrum) and because experimental birds were placed in highly conductive aluminium arenas, which might have disturbed applied radio-frequency fields. Additional experiments with a refined metrological procedure are needed to get better understanding of the effects of electromagnetic noise on the magnetic compass of migratory birds. In particular, it would be instructive to know whether birds are affected by broadband magnetic fields parallel to the geomagnetic fields (for narrow-band fields, this was not the case [2]), and what is the role (if any) of the electric component of the electromagnetic narrow- or broadband fields in disruption of the avian magnetic compass.

Finally, experiments with monochromatic RF fields can present detailed and very valuable information on the spin structure of the receptor, realizing the potentiality of the “behavioural spectroscopy”. However, at the moment too few frequencies have been investigated to evaluate the widths of spin resonances involved. As a result, the sensitivity thresholds obtained for certain discrete frequencies (notably for a “Zeeman resonance” at the Larmor frequency of a free-standing electron spin [3,5]) cannot yet be directly related with corresponding transition rates. Therefore, to estimate whether observed effects can be explained within RPM or the hybrid model, new experimental data on sensitivity thresholds at different frequencies are needed.
